# Universal health coverage and primary care, Thailand

**DOI:** 10.2471/BLT.18.223693

**Published:** 2019-04-01

**Authors:** Kanitsorn Sumriddetchkajorn, Kenji Shimazaki, Taichi Ono, Tesshu Kusaba, Kotaro Sato, Naoyuki Kobayashi

**Affiliations:** a National Health Security Office, 4th Floor, Government Complex Building B, Chaengwattana Road, Laksi, Bangkok, 10210 Thailand.; b National Graduate Institute for Policy Studies, Tokyo, Japan.; c Hokkaido Centre for Family Medicine, Sapporo, Japan.; d Japan International Cooperation Agency, Tokyo, Japan.

## Abstract

Thailand’s policy on universal health coverage (UHC) has made good progress since its inception in 2002. Every Thai citizen is now entitled to essential preventive, curative and palliative health services at all life stages. Like its counterparts elsewhere, however, the policy faces challenges. A predominantly tax-financed system in a nation with a high proportion of people living in poverty will always strive to contain rising costs. Disparities exist among the different health insurance schemes that provide coverage for Thai citizens. National health expenditure is heavily borne by the government, primarily to reduce financial barriers to access for the poor. The population is ageing and the disease profiles of the population are changing alongside the modernization of Thai people’s lifestyles. Thailand is now aiming to enhance and sustain its UHC policy. We examine the merits of different policy options and aim to identify the most promising and feasible way to enhance and sustain UHC. We argue that developing the existing primary care system in Thailand has the greatest potential to provide more self-sustaining, efficient, equitable and effective UHC. Primary care needs to move from its traditional role of providing basic disease-based care, to being the first point of contact in an integrated, coordinated, community-oriented and person-focused care system, for which the national health budget should be prioritized.

## Résumé

La politique de couverture sanitaire universelle de la Thaïlande a bien progressé depuis sa création en 2002. Chaque citoyen thaïlandais a désormais le droit à des services de santé préventifs, curatifs et palliatifs essentiels à tous les stades de sa vie. Néanmoins, à l'instar de ses équivalents dans d’autres pays, cette politique fait face à des difficultés. Un système principalement financé par l'impôt dans un pays où une forte proportion de personnes vit dans la pauvreté devra toujours s'efforcer de limiter l’augmentation des coûts. Des disparités existent entre les différents régimes d’assurance maladie qui fournissent une couverture aux citoyens thaïlandais. Les dépenses nationales de santé sont largement prises en charge par le gouvernement, principalement pour réduire les obstacles financiers qui empêchent les pauvres d'accéder aux services de santé. La population vieillit et le profil des maladies de la population évolue en même temps que les modes de vie des Thaïlandais se modernisent. La Thaïlande a désormais l'intention de renforcer sa politique de couverture sanitaire universelle et d'assurer sa pérennité. Nous examinons les avantages de différentes possibilités d'action et cherchons à identifier la solution la plus prometteuse et réalisable pour renforcer et assurer la pérennité de la couverture sanitaire universelle. Nous soutenons que le développement du système existant de soins de santé primaires en Thaïlande est la meilleure solution pour fournir une couverture sanitaire universelle plus autonome, efficiente, équitable et efficace. Les soins primaires doivent s'écarter de leur rôle traditionnel qui est de fournir des soins de base axés sur une maladie pour être le premier point de contact dans un système de soins intégré, coordonné, orienté vers la communauté et axé sur la personne, ce qui nécessite de donner une priorité élevée au budget national de santé.

## Resumen

La política de Tailandia sobre la cobertura sanitaria universal (CSU) ha progresado mucho desde su creación en 2002. Todos los ciudadanos tailandeses tienen ahora derecho a servicios esenciales de salud preventiva, curativa y paliativa en todas las etapas de la vida. Sin embargo, al igual que sus homólogas en otros lugares, la política se enfrenta a desafíos. Un sistema financiado en su mayoría por impuestos en un país con una alta proporción de personas que viven en la pobreza siempre tendrá que esforzarse para limitar el aumento de los costes. Existen disparidades entre los diferentes planes de seguros sanitarios que ofrecen cobertura a los ciudadanos tailandeses. El gasto nacional en salud lo soporta en gran medida el gobierno, principalmente para reducir las barreras financieras al acceso de los pobres. La población envejece y los perfiles de enfermedad de la población cambian al mismo tiempo que se modernizan los estilos de vida de los habitantes de Tailandia. Tailandia aspira ahora a mejorar y mantener su política de CSU. Se han examinado los méritos de las diferentes opciones de políticas para así identificar la manera más prometedora y factible de mejorar y sostener la CSU. Se sostiene que el desarrollo del sistema de atención primaria de salud existente en Tailandia tiene el mayor potencial para proporcionar una atención primaria de salud más autosuficiente, eficiente, equitativa y eficaz. La atención primaria debe pasar de su función tradicional de proporcionar atención básica basada en la enfermedad a ser el primer punto de contacto en un sistema de atención integral, coordinado, orientado a la comunidad y centrado en las personas, para lo cual se debe dar prioridad al presupuesto nacional de salud.

## ملخص

حققت سياسة تايلند للتغطية الصحية الشاملة (UHC) تقدماً جيداً منذ صدورها عام 2002. حيث أن كل مواطن تايلندي اليوم مخوّل بالحصول على الخدمات الصحية الوقائية والعلاجية والمسكنات الضرورية في جميع مراحل الحياة. ****ورغم ذلك، فكما هو الحال مع السياسات المناظرة في الأماكن الأخرى، فإن هذه السياسة تواجه تحديات. ****إن أي نظام مموّل من الضرائب بشكل أساسي في دولة ذات نسبة عالية من السكان تعيش في فقر، سوف يسعى دائما بكل قوة لاحتواء التكاليف المرتفعة. ****هناك فروقات بين نظم التأمين الصحي المختلفة التي توفر التغطية للمواطنين التايلنديين. ****يقع عبء النفقات الصحية الوطنية بشكل هائل على عاتق الحكومة، وذلك للحد في الأساس من الحواجز المالية بهدف الوصول إلى الفقراء. ****يتقدم السكان في السن وتتغير الخصائص المرضية للسكان إلى جانب التحديث من أنماط حياة الشعب التايلندي. ****تهدف تايلند اليوم إلى تعزيز ودعم سياسة التغطية الصحية الشاملة. ****نحن ندرس مزايا الخيارات المختلفة للسياسة ونهدف إلى تحديد أكثر الطرق الواعدة والفعالة لتعزيز ودعم التغطية الصحية الشاملة. ****نحن نعتقد أن تطوير النظام القائم للرعاية الصحية الأولية في تايلند، ينطوي على أكبر قدر من الإمكانيات لتوفير تغطية صحية شاملة تتميز بمزيد من الاكتفاء الذاتي والكفاءة والإنصاف والفعالية. ****تحتاج الرعاية الأولية إلى الانتقال من دورها التقليدي وهو توفير الرعاية الأساسية بسبب الأمراض، إلى أن تصبح نقطة الاتصال الأولى في نظام رعاية متكامل ومنسق وموجّه نحو المجتمع ويركز على الأشخاص، والذي يجب أن يكون على قمة أولويات ميزانية الصحة الوطنية.

## 摘要

泰国的全民健康覆盖 (UHC) 政策自 2002 年实施以来取得了良好进展。如今，每一位泰国公民都有权终身享受基本预防、治疗和姑息治疗等健康服务。然而，与他国一样，该政策同样面临挑战。在一个贫困人口比例很高的国家，以财政税收资助为主的体系将始终努力容纳不断上升的成本。为泰国公民提供健康覆盖的不同健康保障体系间存在差异。国家卫生支出主要由政府承担，首要目的是为了减少贫困人口获得健康保障的财政障碍。随着泰国人民生活方式的现代化，人口不断趋于老龄化，疾病概况也在发生着变化。泰国目前旨在强化和维持其全民健康覆盖 (UHC) 政策。我们探讨了各种政策方案的优势，旨在找到最具潜力和最可行的方法来强化和维持全民健康覆盖 (UHC) 政策。我们认为，在泰国发展现有的初级卫生保健体系最有可能提供更为自给自立、高效公平和切实有效的全民健康覆盖 (UHC) 政策。在优先考虑国家卫生预算的前提下，初级保健需要从提供基础疾病护理的传统角色转换成综合性、协调性、以社区为导向、关注个人的卫生系统的第一接触点。

## Резюме

С момента внедрения в 2002 году в Таиланде стратегической программы по всеобщему охвату медико-санитарными услугами был достигнут значительный прогресс. Теперь каждый гражданин Таиланда имеет право на получение основных профилактических, лечебных и паллиативных услуг здравоохранения на всех этапах жизни. Однако, как и в других странах, реализация программы связана с проблемами. Преимущественное финансирование из налоговых поступлений в стране, где велика доля бедного населения, всегда означает борьбу с повышением цен. Между разными системами медицинского страхования, охватывающими разные слои тайского общества, существуют значительные различия. Большую часть затрат на национальное здравоохранение несет правительство, чтобы в основном уменьшить финансовые препятствия к получению медицинских услуг бедными слоями населения. Население Таиланда стареет, и профиль заболеваний меняется по мере совершенствования образа жизни в Таиланде. В настоящее время Таиланд ведет целенаправленную политику совершенствования и укрепления стратегической программы по всеобщему охвату медико-санитарными услугами. Авторы изучили достоинства различных вариантов этой программы и задались целью выявить наиболее перспективные и практически осуществимые способы совершенствования и укрепления данной программы. Они доказывают, что развитие имеющейся системы первичной медико-санитарной помощи в Таиланде имеет наибольший потенциал, способный обеспечить более устойчивую, эффективную, равноправную и действенную программу медико-санитарной помощи для всего населения страны. Первичная медико-санитарная помощь должна отойти от традиционной роли обеспечения лечения базовых заболеваний и стать первой точкой контакта в единой, скоординированной, ориентированной на сообщество и на людей системе здравоохранения, создание которой должно стать приоритетным направлением бюджета национального здравоохранения.

## Introduction

Despite sustained periods of political instability^1^ and an under-performing economy,^2^ Thailand’s policy on universal health coverage (UHC) has made good progress since its inception in 2002. Every Thai citizen is now entitled to essential health services at all life stages.^3^ The benefits of the policy comprise essential services in preventive, curative and palliative care for all age groups. Extension of coverage to high-cost services, such as renal replacement therapy, cancer therapy and stem-cell transplants, has improved financial protection for patients.^4^ Well coordinated district health systems enable individuals to seek care or referral at health units close to home.^5^ The resultant increase in service utilization has contributed to a low prevalence of unmet needs for outpatient and inpatient services.^6^

In the decade after UHC was initiated (2001–2011) life expectancy at birth rose from 71.8 to 74.2 years compared with an increase of only 70.3 to 71.8 years during the decade before (1991–2001).^7^ A continuing decline in infant deaths has been recorded from more than 100 per 1000 live births before 1970 to 9.5 per 1000 live births in 2017.^8^ An assessment in the first 10 years of UHC (2001–2010) found reduced out of-pocket spending and fewer households suffering catastrophic spending on health in the poorest and richest quintiles.^9^ Household savings increased among previously uninsured households.^7^ Earlier assessment of the policy noted how expenditure on medicines and medical supplies stimulated the chemical, trade, electricity and transport sectors in Thailand,^10^ reinforcing the argument that investment in health could generate economic returns.^11^ Satisfaction with the policy among providers and seekers of health care have remained consistently high since 2011.^12^ The broad reach of the UHC policy has gained sustained support from the Thai electorate, enabling the policy to succeed through two military governments and seven prime ministers.^13^

Before 2002, Thailand’s health coverage was a patchwork of arrangements for different population groups: the tax-financed civil servants’ medical benefit scheme for public employees; the contributory social security scheme for private employees; the tax-financed medical welfare scheme for people in poverty; and the contributory voluntary health card scheme for households. Taken together, the four schemes should have covered the entire population. However, difficulties assessing the incomes of those informally employed caused the medical welfare scheme to miss its target groups,^14^ while a positive association was found between the presence of illness and the purchase and utilization of the voluntary health card scheme.^15^

The establishment of universal coverage in 2002 enabled the country to provide health coverage to the whole Thai population of 66.3 million persons. The government’s attempt to merge all the schemes was met with resistance from beneficiaries who feared a reduction of their entitlements.^16^ A compromise, once reached, resulted in the national health insurance being overseen by three different schemes: (i) the civil servants’ medical benefit scheme under the finance ministry, covering 5.7 million people; (ii) the social security scheme under the labour ministry, covering 12.3 million people; and (iii) the universal coverage scheme under the public health ministry, covering 47.8 million people or 72% of the population. The universal coverage scheme amalgamated the medical welfare and voluntary health card schemes, while providing a safety net to the residual Thai population attending primary-care units where family physicians acted as gate-keepers to specialty care.

Except for the social security scheme, Thailand’s financing for UHC is predominantly non-contributory, financed by general government taxation. This mode of financing is based on several assumptions:^10^ health insurance premiums are unaffordable to the large numbers of poor people whose need for health subsidies was the reason for the policy in the first place; identifying and collecting premiums from people who should be able to contribute is not logistically straightforward; and increasing the rate of premiums in accordance with rising expenditure could be politically challenging.

Launched when the country was still recuperating from the 1997 Asian financial crisis, the UHC policy was designed to function in difficult financial conditions. Strong social support gave the policy resilience against political and economic challenges.^13^ Nevertheless, unless there is a sustainable approach to lowering the likelihood of medical impoverishment and ill health among the insured, a predominantly tax-financed policy will always struggle to contain challenges to funding the health system. Revenues from taxation are likely to decline; the proportion of working-age citizens peaked in 2010 at 78.9% of the total population (53.0/67.2 million), up from 52.4% (18.8/35.8 million) in 1969.^12^ The continuing rise of the population older than 60 years, an estimated 3.2% (279 000/8 731 419) of whom require constant care, is set to turn the country into an aged society by 2025.^12^ Noncommunicable diseases and modifiable adverse behavioural factors continue to be a burden on people’s quality of life and on health-care costs.^12^ Poor enforcement of road and vehicle safety laws has given Thailand the world’s second highest death rate in road accidents (36.2 deaths per 100 000 people).^17^ Air pollution continues to affect major cities and towns, causing over 48 000 deaths in 2013.^18^ Abnormally wet and dry weather, posing risks to lives and livelihoods, is becoming more severe and frequent.^19^

These challenges place a strain on health-system resources that are the foundation of UHC sustainability.^20^ Sustainability is becoming more important now that Thailand has embedded the sustainable development goals (SDGs) into its 20-year plan for a more inclusive, sustainable and self-sufficient economy. UHC is the target as well as a central pillar of the health-related targets of the SDGs.^21^ Therefore, ensuring that the policy is resilient to adverse financial conditions will be key to achieving the SDGs in Thailand.

As around 6.7 million (10%) of the population are no more than 20% above the poverty line,^12^ Thailand is obliged to continue financing UHC from public money. The limitation is that non-contributory financing via general taxation offers the welfare policy little flexibility to accommodate rising demands in the face of continuing rises in health-care costs. Measures are therefore needed to raise revenue sustainably and use it efficiently, equitably and effectively. In this paper we consider the merits of different policy options and aim to identify the most promising and feasible way to enhance and sustain UHC.

## Raising revenue

With tax revenues contributing around 15% (67 billion United States dollars, US$) of Thailand’s gross domestic product (GDP) of US$ 455 billion in 2017, improving tax collection to 20% of GDP could generate more resources for health.^12^ Yet, expanding the fiscal space in this way does not guarantee greater or consistent funding for health care. One solution is to create new taxes that are earmarked for health spending. Unhealthy products such as tobacco, alcohol and sugary beverages are obvious targets for such taxes. The claim that consumption taxes are regressive (i.e. they take a proportionally greater amount from those on lower incomes) is countered by the findings that poorer people respond more than richer people to a unit change in price.^22^ Since 2001, 2% (US$ 132 million) of the total US$ 6.6 billion of excise taxes on tobacco and alcohol have been earmarked only for health promotion projects and education campaigns in Thailand (i.e. hard earmarking). However, additional revenue from the earmarked tax in a given year should be directed to health-related priorities that promise the most benefits for the money spent (i.e. soft earmarking). The amount and time of the release of the additional allocation can then be at the discretion of the finance ministry, who will balance all competing priorities. The smaller revenue will nevertheless offset the shortfall in government spending.

Charities and partnerships between the public and private sectors are other potential sources of revenue for health. Several hospitals in Thailand have long relied on donations from private businesses and fundraising activities to maintain or upgrade their infrastructure. The most recent example was a 55-day cross-country charity-run by a celebrity in December 2017 to raise US$ 22 million for medical equipment at 11 state hospitals. Nevertheless, such charities can never be the main source of income. Likewise, enhancing public funding with philanthropic funding may sound attractive, but such an option is best suited to shared-value initiatives, such as a government authority collaborating with a private-funded gym to allow a special discount for obese patients.

## Efficient use of revenue

Since resources for health care are never limitless and can never satisfy all possible demands, action is always needed to address rising costs. Two approaches are possible. The first is cost-containment. Being tax-financed, Thailand’s UHC policy is obliged to adopt several strategies to lower excessive spending without lowering net welfare provision.^9^ For example, the government has established a process to assess the merits of high-cost medical advances. The price negotiation working group, under the national essential medicines list subcommittee, has succeeded in bringing down the prices of antiretroviral drugs, intraocular cataract lenses, erythropoietin-stimulating agents and coronary stents, saving the health-care sector an estimated US$ 257 million in 2016. Furthermore, the primary care gatekeeper system, the national formulary and the closed-ended payment system have collectively kept the average health outlay by government at around US$ 167 per capita per year.

The second approach concerns cost-sharing by which patients are required to pay at the point of care, although the available options present difficulties. Cost-sharing applied to the whole population could alleviate the burden on government finances, but could negatively affect the poor, the near-poor and people in vulnerable situations who may be unable to afford services.^23^ The alternative is to limit cost-sharing to a list of supplementary services, but this would likely play a marginal role in lowering health expenditure. Subscribers would mostly be high earners, whereas essential, high-cost services that could impose financial risk to low earners can never be listed in the supplementary category.

## Equitable use of revenue

Any advance towards efficiency and equity gains will always be weakened by the existence of disparate national insurance schemes in need of a unifying mechanism. The Thai national health insurance is fragmented by the three main schemes whose eligibilities are linked to employment status. Although non-competing, each scheme operates under its own legal framework. The inevitable disparities mean that not all groups of the population have equal access to similar packages of health care. Amalgamating the schemes requires high-level action which, given the vested interests of beneficiaries,^16^ is politically sensitive and challenging. However, integration may not be as important as ensuring that all schemes offer the same services with similar purchasing arrangements for services. A recent effort to equalize different statutory schemes via fixed fees for emergency health care could be a model for other services.

## Effective use of revenue

Under the UHC policy, services are offered that are deemed to be cost–effective, beneficial for the worse-off and protective against impoverishment to households.^24^ Regional administrations and local health-care facilities in Thailand have the flexibility to align services with the preferences of the community. Yet the focus has been on eliminating and controlling specific illnesses, rather than improving the coordination and responsiveness of the integrated care process. Most notably, increased utilization of comprehensive services together with financial risk protection may have steadily lowered all-cause mortality, but the prevalence of many manageable conditions such as diabetes and tuberculosis are not going down.^9^ Success of UHC depends on health-care delivery to improve the well-being of citizens in a way that is efficient for the country. While providing care to the whole population should not lead to government bankruptcy, delivering sub-standard services can also be a burden on public finances.^25^ Focusing on disease processes without consideration of the contexts in which people live, work and cope with their co-existing illnesses is unlikely to provide the clinician with the complete picture of the problem.^26^ Attention to the patient’s problems is as important as attention to their diagnoses. Thus, the quest to deliver value for money could best be led by people-centred primary care.^26^^,^^27^

## Enhanced role for primary care

Despite efforts to ensure that Thai citizens will not be financially ruined by needed services, the UHC policy is facing challenges. First, although financing through general taxation is currently the most equitable and efficient way of paying for health care, the cost of the policy (US$ 14 809 million; 17% of the total US$ 89 415 million government expenditure in 2017) is one of the highest among low-and middle-income countries.^13^ Second, although attempts have been made to control costs, rising health-care costs will always be an issue owing to the growing health needs and expectations of the population and increasing costs of technological and medical advances. Third, the reality of the legal framework governing each funding pool makes it likely that the current multi-tier system, which has no unifying mechanism to control expenditure, will continue for a long time. Fourth, the prevalence of preventable and controllable infectious and non-infectious illnesses, such as diabetes mellitus, hypertension, renal failure, tuberculosis and human immunodeficiency infection, is showing an upward trend.^9^ Identifying and mobilizing the necessary resources, controlling excessive spending and equalizing payment methods, without ensuring improved health, would be a wasted investment.

Overcoming these challenges, separately or in combination, could add strength and endurance to the UHC policy. The interconnectedness of these challenges is such that a solution may be found that can improve quality of care without undermining the efficiency and equity of the policy. A robust primary care system can manage acute, chronic and social conditions affordably and effectively and could be the answer to both controlling costs and improving people’s health and well-being.^28^

Table 1 summarizes key options for Thailand’s UHC sustainability and the all-encompassing potential of primary care to address those challenges.

**Table 1:** The role of primary care in enhancing and sustaining universal health coverage in Thailand

Measure	Current situation	Potential of primary care	Enhanced role for primary care
Sustainable raise of revenue	Funding UHC through levying tax contributions on the population is hampered by the difficulty of determining incomes accurately among the self-employed. Even if incomes could be determined, contributions could be onerous for non-poor low earners. General taxation is the most equitable way to fund UHC. Yearly rises in health-care expenditure can be supplemented by earmarked taxes on products that are damaging to health and by boosting public finance with that of local health authorities or the private sector	Self-sustaining and diverse care	Primary care provides improved accessibility, continuity, coordination and comprehensiveness of care. Improving health services builds public trust that provides political support for a tax-funded policy. Involving the community empowers them to address health issues that affect them. Alliances across different non-health sectors, e.g. business owners, nongovernmental organizations and religious communities, can nurture robust funding, best thinking, and innovation that can be beneficial to individual health and community resilience
Efficient use of revenue	Future UHC costs can be contained by factors such as central procurement, enforced use of the national formulary, assessment of the merits of new medical interventions, designation of family physicians as the gatekeepers of access to specialist care, and use of closed-end payments. Cost-sharing could reduce excessive demand for free-of-charge care, but the adverse effect on the poor would defeat the aim of UHC. A two-tier health benefit system, in which voluntary contributions for supplementary benefits are paid out-of-pocket, would play a marginal role since subscribers will mostly be high earners whereas essential, high-cost services that could impose financial risk to low earners can never be listed in the supplementary category. The existence of multiple national health insurance schemes with no conformity of benefits and payments tends to dilute cost-control effects. Rising health costs for government will continue to be an issue due to greater health needs and rising expectations of the population and increasing high-cost, but necessary medical interventions	Affordable care	Primary care lowers the costs of health services through cost–effective preventive health care and deploying family physicians to lead medical teams. Primary care provides coordination, continuity and comprehensiveness of care, leading to greater efficiency and better health outcomes. Primary-care teams treat a heterogeneous group of patients and can design a process of care that will allow them to match patients’ needs with the available health-care resources
Equitable use of revenue	Fragmentation across national insurance schemes with different benefit packages and payment mechanisms creates disparities in service provision across groups of the population. Given the vested interests and legal framework governing each funding pool, total integration of all schemes is politically challenging. Integration is being trialled in emergency-care services via a fixed-fee schedule which could further defragment other services, such as disease prevention and chronic care	Available care	A system based on primary care is more likely to achieve conformity of essential benefits across the different national health schemes in Thailand. Frontline services are accessible by beneficiaries of all schemes and should narrow socioeconomic disparities across schemes. Thailand has already seen evidence of a marked improvement in under-five mortality across income quintiles after establishment of the primary system. Primary care is also well placed to address the social determinants of health, thereby minimizing health inequities
Effective use of revenue	The health benefits provided under UHC must be cost–effective, beneficial to the worst-off groups and protective against impoverishment of households. The focus of care has been on eliminating and controlling specific illnesses, rather than improving integrated care and understanding the contexts in which people live. Although all-cause mortality shows a steady decline in Thailand, the prevalence of preventable and controllable illnesses is rising	Holistic care	Primary care prioritizes preventive and promotive care as a complement to standard curative and palliative care. Primary care provides more comprehensive and better coordinated care over time, allowing providers to recognize and meet patients’ physical, emotional and social needs, and improve their journeys through the health-care system

UHC: universal health coverage.

### Affordable care

The strength of primary care rests on its characteristics of accessibility, continuity, coordination and comprehensiveness.^28^ When all these dimensions are strengthened,^29^ primary care has been shown to improve the patient’s journey through the health system at a lower cost than specialty-oriented care.^30^ The combined effect of these characteristics improves the cost–effectiveness and efficiency of the system and the health of patients in several ways: by designing the most appropriate clinical pathways for acute, chronic and ambulatory conditions; by matching patients’ needs with the available health-care resources; and by enhancing the system’s ability to adapt to new circumstances.^31^ Consequently when family medical teams are led by primary-care physicians, costs tend to fall and patients’ health improves.^32^

### Available care

Achieving conformity of benefits across different national health insurance schemes could be achieved by promoting primary care as the first point of contact with the health service. This makes all essential services accessible by beneficiaries of all schemes, while the community-wide reach of such a system could narrow the gaps between rich and poor in access to care.^33^ If the social determinants of health are taken into account, health inequities can be minimized further.^34^ Reforms to the primary-care system in Thailand from the 1970s through to the 1990s, including large investments in infrastructure development and workforce retention in the community, were followed by a marked improvement in under-five mortality across income quintiles.^35^ Investments in primary care thus deliver greater equity than investments in the health-care system in general.^36^

### Holistic care

Spending on preventive and promotive care, as a complement to curative and palliative services, is prioritized in the primary-care setting.^28^ More comprehensive services allow providers to better meet the needs of patients with multimorbidities.^37^ Better coordination facilitates patients’ navigation through the health-care system.^38^ Primary care addresses the patient’s physical, emotional and social needs. Such person-focused care over time provides better recognition of patients’ health problems.^39^

### Self-sustaining care

Improved quality of care via an enhanced primary care system would help to build trust and solidarity from the public, who are accustomed to seeking care at hospitals and private practices. Building credibility will provide political support for UHC and hence stability of funding for services financed by taxes paid mostly by the middle classes.^40^ Perceptions of better care and safety could turn these financially secure groups into strong supporters of the policy and vigorous advocates for better services.^40^

### Diverse care

Despite the advantages of a primary-care system, it has been noted that orientation of national policies and practice towards primary care does not necessarily guarantee better health for the population.^41^ This constraint has been attributed to factors such as high unemployment, high rates of smoking, heavy alcohol drinking, social inequality and diets high in saturated fat.^41^ Many factors go beyond the immediate scope of health care: for example, when people live in neighbourhoods where they cannot get fresh produce, have no safe, green spaces for exercise, and have no incentives to exercise.^34^^,^^42^^,^^43^ UHC needs to progress beyond a focus on treating diseases by ensuring the adequacy of primary-care services in addressing people’s necessities.

### UHC sustainability and SDG achievement

**Fig. 1:**
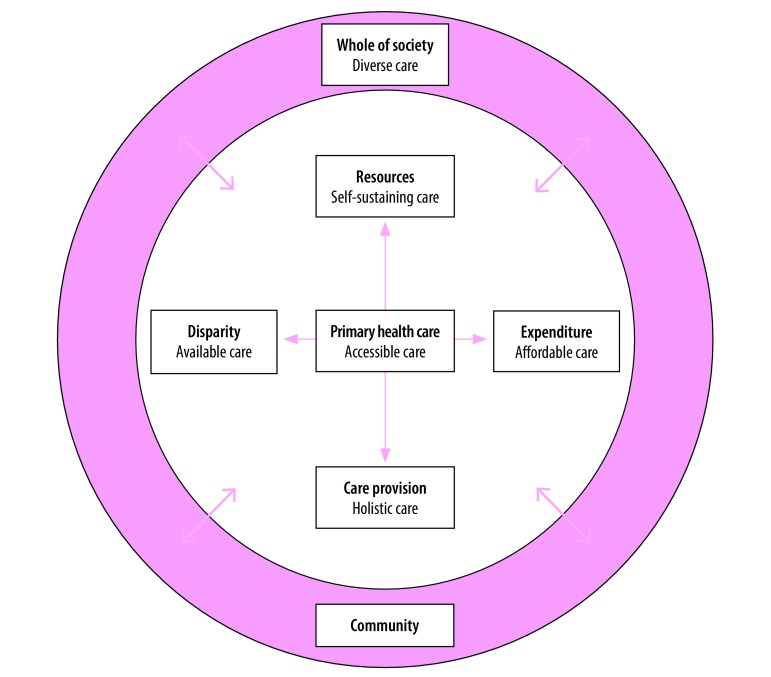
The potential of primary care for achieving sustainability of Thailand’s universal health coverage policy

Note: As a bridge between health-care sector and the community, primary care enhances the financing and service delivery of the universal health coverage through the provision of people-centred care that is affordable, available, accessible and self-sustaining.

Operating at the intersection of health care and community, primary care pays attention to a diversity of issues affecting the well-being of the population (Fig. 1).^27^^,^^43^ Primary care empowers and enlists the community to tackle wide-ranging socially determined health issues.^44^^,^^45^ Such care nurtures participatory governance, social cohesion and health literacy, as well as paves the way for an alliance across the public and private health sectors, and the population. From such alliances arise collective leadership, coordination across organizations and strong infrastructure. This whole-of-society approach embraces the diverse skills, robust connections and social support that can strengthen individuals’ health and community resilience.^46^ The successful rescue of a football team from a cave in northern Thailand in July 2018^47^ is a recent example of how the combined force of technical power, political power and operational power is crucial to solving or averting a major disaster, minor crisis or daily inconvenience affecting a community.

## Next steps for UHC

Several measures to enhance and sustain Thailand’s UHC policy exist: increasing revenue; optimizing use of resources; reducing differentials across health insurance schemes; and improving quality of care. With the support of specialty services,^48^ people-centred primary care stands out for its beneficial effects on health outcomes, on community resilience and on the economy.^43^ As the most pragmatic measure to cultivate health-system resilience for UHC sustainability, strengthening primary care will have a valuable and sustainable impact on health-system performance and people’s health.^49^

Primary care has been central to Thailand’s health-care reform efforts since the 1970s, through national policies that expanded the numbers of health facilities and health workforce and extended financial coverage to all parts of the country.^5^^,^^50^ However, the system has to move from its traditional role of providing basic disease-based care to being the first point of contact in integrated, coordinated, community-oriented and person-focused care for which the national health budget should be prioritized. 

Primary care has the potential to provide affordable care, enhance the quality of care, level disparities across different groups, mobilize non-public financial resources and rally non-health sectors for social and individual good. Developing the health system with a focus on primary care will enhance and sustain Thailand’s UHC policy and, in synchronizing health and social care,^27^^,^^43^ be a crucial component towards achieving the SDGs embedded in the national agenda.
